# Beyond the Imposter: Deciphering Capgras Syndrome and Multidisciplinary Management

**DOI:** 10.1155/crps/5541100

**Published:** 2025-08-11

**Authors:** Manuel Glauco Carbone, Tommaso Merati, Rossella Miccichè, Camilla Callegari, Beniamino Tripodi, Claudia Tagliarini, Icro Maremmani, Donatella Marazziti

**Affiliations:** ^1^Department of Medicine and Surgery, Division of Psychiatry, University of Insubria, Varese, Italy; ^2^Saint Camillus International University of Health and Medical Sciences, Via di Sant'Alessandro 8, Rome 00131, Italy; ^3^Department of Mental Health and Addictions, Division of Psychiatry, ASST Crema, Crema, Italy; ^4^Department of Mental Health, Psychiatric Diagnosis and Treatment Service, Sant'Elia Hospital, ASP 2, Caltanissetta, Italy; ^5^Department of Clinical and Experimental Medicine, University of Pisa, Pisa, Italy

**Keywords:** Capgras syndrome, delusional belief, Lewy body dementia, misidentification, salience

## Abstract

Capgras syndrome (CS) presents a unique diagnostic and management challenge, particularly when associated with neurodegenerative conditions. This case report describes a 73-year-old female diagnosed with Lewy body dementia (LBD) who developed CS, manifesting as the delusional belief that her deceased husband had been replaced by an imposter. The clinical presentation was complex, including pronounced sleep disturbances, cognitive decline, behavioral anomalies, and visual hallucinations, necessitating a thorough differential diagnosis process. We detail the patient's history, clinical findings, and the investigations undertaken, including brain imaging and cognitive assessments. The management approach involved a personalized treatment plan integrating Lithium sulfate, Citalopram, and Lurasidone, alongside psychosocial interventions. While a partial improvement was observed, the case underscores the complexities of managing CS in the context of LBD, highlighting the need for tailored and multidisciplinary approaches to care. This case contributes to the limited literature on CS in LBD and provides practical insights for clinicians managing similar presentations.

## 1. Introduction

Capgras syndrome (CS), also known as “imposter syndrome” or “illusion of doubles,” is a rare neuropsychiatric condition in which an individual develops the delusional belief that a familiar person, often a family member or close friend, has been replaced by an identical imposter [1].

This inherently paradoxical belief persists despite recognizing the person's physical and behavioral traits, highlighting a profound dissociation between the processes of recognition and identification. From a psychopathological perspective, CS falls within the spectrum of delusional disorders, particularly among bizarre delusions, which are those that are patently absurd and incomprehensible in light of the individual's cultural context [2].

CS is most commonly associated with schizophrenia, delusional disorder, and schizoaffective disorder [3, 4]. In schizophrenia, as described by Edelstyn and Oyebode, CS often appears as part of a broader delusional framework. The impaired thinking and difficulty distinguishing reality often seen in schizophrenia can contribute to the development of this specific delusion [5]. Delusional disorder is characterized by persistent, fixed delusions, and CS fits this description perfectly, often emerging as the primary symptom [6]. Schizoaffective disorder combines features of both, schizophrenia and mood disorders. Similar to schizophrenia, the distorted thinking and reality testing in schizoaffective disorder can lead to the emergence of CS [7].

The onset of CS can be acute or insidious, meaning it can appear suddenly or develop gradually over time. It can also be transient or chronic, meaning it can be temporary or long-lasting. The course of CS is highly variable and depends on the underlying cause. In cases associated with treatable medical conditions, addressing the underlying condition may lead to the resolution of the delusion. However, in cases associated with neurodegenerative disorders or severe psychiatric illnesses, the syndrome may persist and fluctuate in intensity over time, with periods of remission and relapse [8–11].

The syndrome's clinical presentation often includes, a range of affective and behavioral disturbances. Patients may exhibit emotional detachment or apathy towards the perceived imposter, coupled with heightened anxiety, fear, and paranoid ideation. These emotional responses can manifest in behavioral changes, including avoidance of the “imposter”, agitation or aggression in their presence, and attempts to protect themselves from the perceived threat. Additionally, individuals with CS may present with comorbid psychopathological symptoms, such as generalized anxiety, tension, hyperarousal, and other psychiatric manifestations [6]. It is crucial to acknowledge the heterogeneity of CS, as the specific constellation and severity of symptoms can vary significantly across individuals. This variability underscores the complexity of the disorder and the need for comprehensive clinical assessment to accurately diagnose and manage CS.

Accurately diagnosing CS poses a significant challenge for clinicians due to the considerable overlap with symptoms of other psychiatric and neurological disorders. As previously mentioned, delusional misidentification, a hallmark of CS, is not pathognomonic and can manifest in various other clinical conditions (e.g., schizophrenia, delusional disorder, neurodegenerative disorders). Therefore, a comprehensive and meticulous clinical evaluation is paramount for accurate diagnosis.

This case report presents the case of a 73-year-old female patient who presented with symptoms consistent with CS, accompanied by significant mood instability. Clinical and historical evaluations suggested a potential underlying neurodegenerative pathology, specifically Lewy body dementia (LBD). A comprehensive treatment plan, incorporating mood stabilizers, antidepressants, and antipsychotics, was implemented. This multifaceted approach resulted in a gradual, progressive, though partial, improvement in the patient's overall clinical presentation.

## 2. Case Presentation

### 2.1. Sociodemographic Details

The patient is a 73-year-old Caucasian female who previously held administrative and clerical roles. Her career included several years as a secretary and/or bookkeeper for various private firms, culminating in over 15 years as a clerk at a single company. The patient lived independently for most of her life. However, for the past 3 years, she has resided with her daughter and son-in-law, who provide daily support and manage her healthcare needs. Notably, she completed high school and some college courses, suggesting a previously higher level of cognitive function.

### 2.2. History of Present Illness

Over the past year, she developed progressive symptoms, starting with sleep disturbances marked by fragmented sleep and frequent awakenings, along with daytime somnolence and cognitive fluctuations.

More recently, she exhibited behavioral and cognitive changes, notably Capgras delusion, a firm belief that her deceased husband, who died 5 years ago, has been replaced by an imposter. She recognizes his death but believes this imposter, described as slightly taller with a deeper voice, has taken his place. The delusion is persistent and causes her significant distress, with anxiety and confusion about his presence in her home. She feels constantly on edge and unable to relax.

As in CS, the patient lacks awareness of the delusional nature of her beliefs and becomes agitated when challenged. Although, she experiences visual hallucinations, it's important to distinguish these from the Capgras delusion, which involves misidentifying a real person as the imposter, rather than hallucinating figures. Further, there are no associated persecutory delusions or auditory hallucinations related to the identified imposter. However, she reported seeing face-less figures or strangers mainly during sleep transitions (hypnagogic and hypnopompic hallucinations), which are considered separate from her delusion.

The progressive nature of her condition is further illustrated by increasing forgetfulness, difficulty misplacing items, and impaired recall of recent events. These cognitive deficits, coupled with the fixed delusion and earlier sleep disturbances, suggest a complex and evolving neurological condition. The specific focus on her deceased husband suggests a strong emotional component to her delusion.

### 2.3. Physical Examination

The neurological examination revealed subtle findings potentially suggestive of parkinsonism, a common feature in LBD [12]. The patient had slight resting tremors in both hands and moves a bit slowed. Her gait was slightly unsteady, with a shuffling walk, supporting this possibility. However, other parts of the exam were normal: Romberg's sign and Mingazzini test were negative, indicating good balance and proprioception. Also, cerebellar function and cranial nerves were normal. Importantly, there was no recent head trauma, ruling out injury as a cause.

### 2.4. Mental Status Examination

The patient presented as alert and lucid during the examination, with intact remote memory as confirmed by both the patient and her daughter. However, concerns were raised regarding recent short-term memory difficulties and instances of forgetfulness. To further assess these concerns, a series of cognitive tests were administered, including the clock drawing test, geometric figure copying, digit span tasks, verbal fluency, and short-term memory recall. These tests revealed impairments in attention, short-term memory, and executive function, consistent with the cognitive profile often observed in a neurodegenerative disorder. She maintained eye contact intermittently, notably becoming evasive when topics related to her delusional beliefs were explored.

The patient appears somewhat guarded. Her slightly stooped posture and reduced spontaneous movements suggest mild psychomotor slowing. The patient's personal hygiene and grooming appear appropriate. Her speech was globally fluent but sometimes hesitant, with occasional word-finding difficulties. Her mood is subjectively reported as “anxious”. She exhibited emotional lability, irritability, and nervousness. Although, the patient reported a subjective experience of mildly decreased energy, a comprehensive assessment of her motivation and volition proved challenging due to her persistent preoccupation with her delusional beliefs. Her thought process was circumstantial, with a tendency to perseverate on the delusion that her husband is an imposter. Importantly, she showed no thoughts of suicide or violence. During the exam, she did not have persecutory delusions or ideas of reference related to the impostor. Her anxiety and distress mainly stemmed from the misidentification and the disruption it caused to her sense of familiarity and security, rather than fears of harm or malevolent intent from the imposter. Her thought speed was normal.

She reports increased anxiety throughout the day, especially in the evening, with restlessness, hand-wringing, and repeatedly asking about her husband's whereabouts. She has a decreased appetite, occasional nausea (mainly in the morning), and has lost about 5 kg in the past year. She has difficulty falling asleep, wakes frequently at night, and has vivid, often disturbing dreams that she sometimes struggles to distinguish from reality. These symptoms support a suspected diagnosis of REM sleep behavior disorder. She also reports visual hallucinations, figures without faces or unfamiliar people, that mostly occur during sleep transitions (hypnagogic and hypnopompic).

### 2.5. Psychiatric and Medical History

Around age 70, the patient developed depression and panic symptoms, even though there were no recent stressful events. These symptoms appeared about 2 years after her husband's death, suggesting a delayed emotional reaction. She experienced low mood, lack of motivation and interest, loss of appetite with stomach issues, preoccupation with bowel movements, and fear of constipation, mood swings, irritability, fragmented sleep with frequent awakenings, agitation upon waking, and anxiety related to her heart and breathing, mainly in the evening. Her doctor prescribed paroxetine 20 mg/day and alprazolam 1 mg/day, which she took for about a year with improvement. She then stopped the medications on her own without symptoms returning.

Over the last year, her daughter noticed changes in her sleep. She started napping often during the day and waking confused at night. She resumed taking alprazolam, but it increased her daytime sleepiness. On her daughter's advice, she began taking 2 mg/day of extended-release melatonin, which slightly improved her sleep. This disrupted sleep pattern, with excessive sleepiness during the day and confusion at night, suggests REM sleep behavior disorder, a sign of LBD [13]. She also reports hypnagogic and hypnopompic misperceptions.

Her medical history includes hypertension, high cholesterol, and osteoarthritis, all under treatment. She has no history of stroke, head injury, seizures, or other neurological issues that could explain her current symptoms.

### 2.6. Substance Use Disorder History

The patient denies any history of alcohol, tobacco, or illicit drug use. This is important to establish, as substance-induced psychosis can sometimes present with delusional symptoms, including Capgras-like delusions [14]. Ruling out substance use as a contributing factor strengthens the likelihood that her symptoms are due to an organic cause, such as DLB [15].

### 2.7. Family History

A thorough family history was obtained to assess potential genetic predispositions to the patient's current condition. There is no known family history of dementia or other neurodegenerative disorders. The patient's mother reportedly experienced significant anxiety throughout her life, although, she never sought formal treatment. It is important to note that family history of psychiatric conditions, while not directly diagnostic of a specific disorder in the patient, can be informative [15].

Further investigation into the nature and potential genetic basis of the mother's anxiety could be considered, as some psychiatric conditions may share genetic risk factors with neurodegenerative disorders [16].

### 2.8. Investigations

In the following months, several tests were done. A brain CT scan a few days after the initial exam showed mild, diffuse brain shrinkage, slightly below normal for her age. There was some possible volume loss in both temporal lobes, especially in the hippocampi and amygdalae, but this is nonspecific. Slight enlargement of the frontal horns of the lateral ventricles suggested mild frontal atrophy, mainly in the dorsolateral prefrontal area. No signs of bleeding, mass, or midline shift were seen.

Brain MRI, acquired approximately 1 month after the initial clinical assessment, demonstrated mild to moderate generalized cortical atrophy. The atrophy was most pronounced bilaterally in the temporal lobes, particularly affecting the hippocampi and amygdalae. Frontal lobe atrophy, predominantly involving the dorsolateral prefrontal cortex, was also observed. Notably, there was no evidence of acute infarction, hemorrhage, or space-occupying lesions, effectively excluding alternative diagnoses, such as stroke or tumor.

To further evaluate, the possibility of LBD or Parkinson's disease (PD) dementia, a dopamine transporter scan was ordered. This imaging modality can detect dopamine transporter deficiencies, a common hallmark of the disease [17]. However, the SPECT DaT scan, performed approximately 10 days after the MRI, did not reveal any lesions indicative of dopaminergic deficits.

Formal neuropsychological testing was recommended to obtain a comprehensive assessment of the patient's cognitive functioning, but she declined, potentially indicating a lack of insight into her condition, a feature sometimes observed in neurodegenerative disorders [1]. Blood tests, including a complete blood count, comprehensive metabolic panel, thyroid function tests, and vitamin B12 levels, were all within normal limits, ruling out common reversible causes of cognitive impairment and reinforcing the suspicion of a primary neurodegenerative disorder [10] (Table 1).

### 2.9. Diagnosis

#### 2.9.1. Provisional Diagnosis

The constellation of clinical presentations, patient history, and preliminary investigations suggests a provisional diagnosis of a neurodegenerative disorder, with LBD being the most probable consideration [18]. This diagnosis is supported by the following factors:

• Clinical onset: Although, a definitive diagnosis requires neuropathological confirmation, the patient's insidious clinical presentation, primarily characterized by prominent delusional misidentification, pointed towards a neurological etiology. This suspicion was further supported by the patient's age and unremarkable psychiatric history. Additionally, the clinical course effectively ruled out acute neurological events.
• Cognitive fluctuations: The patient exhibits significant fluctuations in her cognitive abilities, including periods of confusion and disorientation. While such fluctuations can be observed in various forms of dementia, they are particularly characteristic of LBD.
• Visual hallucinations: The presence of complex visual hallucinations, specifically manifesting as Capgras delusion in this case, is a significant feature often associated with DLB and may appear early in the disease course, although, not exclusively.
• Parkinsonism: The patient exhibits mild parkinsonian symptoms, including slow movements and gait disturbances, which are consistent with the motor features frequently observed in DLB.
• Behavioral and psychological symptoms: The early and prominent presence of behavioral and psychological symptoms, such as anxiety, agitation, and delusions, strongly suggests DLB, as these symptoms often precede significant cognitive decline in this condition.

Furthermore, the patient's absence of a history of visual disturbances supports CS as the underlying mechanism for her delusional misidentification. This distinction is crucial, as other delusional misidentification syndromes, such as Fregoli syndrome, are often associated with visual impairments [18]. It is important to acknowledge that the absence of dopaminergic deficits on SPECT DaT scan, while reducing the likelihood of PD dementia, does not entirely exclude LBD, as the sensitivity of this test is limited, particularly in early stages of the disease.

#### 2.9.2. Differential Diagnoses

While LBD is the most likely diagnosis based on the available evidence, it is crucial to consider and exclude other conditions with overlapping clinical features. Although, memory impairment is present, the prominence of visual hallucinations, parkinsonism, and cognitive fluctuations, particularly early in the disease course, argue against a primary diagnosis of Alzheimer's disease (AD). In this condition, memory impairment typically predominates the clinical presentation, with other cognitive and behavioral symptoms emerging later [10]. Distinguishing DLB from PD dementia hinges on the temporal relationship between cognitive and motor symptom onset. In this case, the patient's cognitive and behavioral symptoms appear to have emerged first, favoring a diagnosis of DLB [9].

However, further observation and longitudinal assessment are necessary to confirm this distinction, as the clinical presentations of both conditions can evolve over time, along with consideration of alternative diagnostic markers and potential future imaging studies, to more definitively confirm this distinction.

Moreover, although memory impairment is more typically associated with AD, it can also be present in DLB, though often with different characteristics, such as greater fluctuations.

### 2.10. Assessments

A comprehensive assessment of the patient's cognitive and functional status was conducted, revealing significant impairments impacting her daily life.

#### 2.10.1. Cognitive Assessment

Formal neuropsychological testing was offered to obtain a detailed understanding of the patient's cognitive profile; however, she declined to participate. Despite this limitation, bedside cognitive assessments were administered, revealing deficits suggestive of impairments in attention, executive function, visuospatial abilities, and memory. These findings align with the cognitive profile frequently observed in individuals with LBD [10].

#### 2.10.2. Functional Assessment

A functional assessment was conducted to evaluate the impact of the patient's cognitive impairments on her ability to perform daily activities. The assessment revealed that the patient now requires assistance with various instrumental activities of daily living (IADL), including managing finances, preparing meals, and using transportation. Additional IADLs requiring assistance include medication management (remembering to take medications as prescribed and managing refills), housekeeping (maintaining a safe and clean-living environment) and shopping (planning and purchasing groceries and other necessities). However, she remains independent in basic activities of daily living, such as bathing and dressing. This decline in functional capacity, specifically in IADLs, aligns with the progressive nature of DLB and significantly impacts both the patient's quality of life and the caregiver burden [1].

#### 2.10.3. Caregiver Burden Assessment

Recognizing the significant impact of DLB on caregivers, an assessment of caregiver burden was conducted with the patient's daughter. The assessment revealed that she is experiencing substantial stress and strain related to her mother's care needs. This finding underscores the importance of providing comprehensive support and resources to caregivers of individuals with DLB, as their well-being is paramount in maintaining the patient's quality of life and ensuring appropriate care [1].

#### 2.10.4. Functional Decline

It is important to note that prior to the onset of her current symptoms, the patient was fully independent in all aspects of her daily life. The observed decline in functional abilities, necessitating assistance with previously manageable tasks, such as medication management, further supports the presence of an underlying neurodegenerative process [15]. This deterioration highlights the pervasive impact of her condition on her daily life and emphasizes the need for ongoing monitoring and support.

### 2.11. Treatment Details and Clinical Course

#### 2.11.1. Medications at the Treatment Entry

At the treatment entry, the patient was taking the following medications: hydrochlorothiazide 25 mg daily for hypertension, atorvastatin 20 mg daily for hyperlipidemia, acetaminophen 500 mg as needed for osteoarthritis pain, melatonin at bedtime for sleep (prescribed by the general practitioner).

#### 2.11.2. Treatment History and Response

Approximately 2 months before the referral, her general practitioner prescribed Quetiapine 50 mg/day for sleep and agitation, but it was ineffective. Due to lack of improvement and her mother's leg weakness and drowsiness, her daughter, with the general practitioner, discontinued it.

Given, her initial sleep issues and hesitation towards medications, a careful approach was chosen. Following a thorough evaluation, of her clinical history, psychopathological profile, and the predicted course of her neurodegenerative condition, treatment was initiated with Lithium sulfate 83 mg/day, Citalopram 10 mg/day, and Lurasidone 37 mg/day.

This multifaceted approach was carefully tailored to address the complexity of her symptoms. Lithium was included primarily for its mood-stabilizing properties, aiming to mitigate the patient's anxiety, agitation, and sleep disturbances, which were believed to exacerbate her delusional symptoms. Its selection was also based on its potential to provide a degree of neuroprotection, as suggested by research in similar neurodegenerative contexts [19–23]. Citalopram was added to address depression and anxiety, and Lurasidone to control psychosis and agitation while limiting motor side effects. The treatment was tailored to her needs, with close monitoring for adverse effects.

This combination strategy was thus designed to offer a comprehensive and balanced intervention, addressing the patient's multifaceted needs while rigorously monitoring for any potential adverse reactions, given the inherent vulnerability associated with her condition.

At the subsequent visit, after about 1 month, she showed overall improvement: mood slightly better, less anxiety, and sleep problems. Her delusions persisted but caused less distress. She resumed cooking despite ongoing cognitive deficits. Based on her progress, Lurasidone was increased to 74 mg/day.

3 months later, she continued to improve: increased activity levels, improved concentration and attention, less anxiety, more regular sleep, fewer episodes of confusion and less intense emotional response to delusional thoughts. However, her motor skills slightly declined, with slower gait and worsened tremors. Cognitive domains also remained partially impaired. Given these results, the current treatment was maintained with close monitoring (Table 2).

Given the inherent risks associated with lithium use, especially in geriatric patients, meticulous and routine monitoring of lithium levels, as well as renal and thyroid function, was implemented throughout the treatment period. Serum lithium levels were evaluated weekly during the first month, and subsequently biweekly, to maintain concentrations within the narrow therapeutic window of 0.4–0.8 mEq/L, thereby minimizing the potential for toxicity. To proactively identify early indications of renal or thyroid dysfunction, renal function, including serum creatinine and estimated glomerular filtration rate (eGFR), and thyroid function, encompassing TSH and free T4 levels, were assessed on a monthly basis.

## 3. Discussion

CS, though rare, is a challenging diagnosis for clinicians. This is because it can occur in many different conditions, including psychiatric disorders, neuropsychiatric illnesses, neurodegenerative diseases, and neurological conditions. It is characterized by a persistent delusional belief that a close person, often a family member, has been replaced by an imposter, because of its complexity, a careful and thorough evaluation is essential to establish the correct diagnosis and develop an effective treatment. Misdiagnosis can lead to inappropriate or harmful interventions. Therefore, understanding the diverse causes and presentation of CS is crucial for clinicians to provide the best care for patients with this confusing condition.

In this case, a 73-year-old woman was diagnosed with CS. Over 6 months, she had a persistent delusion that her husband, who died 5 years earlier, had been replaced by an imposter. Initially, her belief was intermittent, but it became continuous and caused her and her family significant distress. She showed no insight into her delusion and became more agitated when questioned. The fact that she also had a depressive episode 2 years after her husband's death, along with her delusional focus on him, suggests that her grief and emotional attachment to her husband heavily influenced her mental state. This strong emotional connection highlights how grief can have lasting effects on her psychological health.

In addition to the prominent Capgras delusion, the patient presented with a constellation of significant symptoms, painting a broader picture of her condition. For approximately 1 year prior to the diagnosis, she had been experiencing sleep disturbances, with frequent nighttime awakenings, excessive daytime sleepiness, and confusion during the day. These issues could be suggestive of an underlying neurological or neurodegenerative process [13]. Furthermore, the patient exhibited signs of cognitive decline, such as increasing forgetfulness, difficulties recalling recent events, and challenges in locating misplaced items, which further pointed towards a potential neurodegenerative etiology [24]. Significant behavioral changes were also observed, including irritability, nervousness, and anxiety, especially in the evening hours. These changes could be attributed to the distress caused by her delusional beliefs, sleep disturbances, or underlying neurological changes [25]. The patient also reported physical symptoms, such as decreased appetite, morning nausea, and unintentional weight loss (~5 kg over the past year). While nonspecific, these somatic symptoms warranted further investigation to rule out potential medical contributors [26]. Finally, the patient described experiencing hypnagogic and hypnopompic hallucinations, a pattern frequently associated with neurodegenerative disorders, such as LBD [27].

The convergence of these symptoms, coupled with the patient's history and clinical findings, guided the diagnostic algorithm.

The late onset of depressive and panic-agoraphobic symptoms around the age of 70 suggests the emergence of a neurodegenerative process, is significant in the context of her otherwise unremarkable psychopathological history until that age, rather than solely indicating a vulnerability to psychiatric illness [28, 29]. These symptoms should be considered initial neuropsychiatric manifestations of the underlying neurodegenerative disorder.

The patient's current clinical presentation reveals a complex interplay of symptoms that strongly suggest an underlying neurodegenerative disorder. The presence of Capgras delusion, sleep disturbances, cognitive impairment, behavioral changes, somatic symptoms, and visual hallucinations necessitates a careful differential diagnosis, with LBD and frontotemporal dementia (FTD) emerging as the primary considerations. The prominent visual hallucinations and sleep disturbances align with disruptions frequently observed in DLB [30]. While the behavioral variant of FTD remains a possibility, it is less likely given the absence of prominent behavioral and language disturbances typically associated with this condition [31, 32].

Clinical assessment and physical examination noted the presence of findings potentially suggestive of parkinsonism, including mild resting tremors, slowed movements, and a mildly unsteady gait. These subtle parkinsonian features further support the possibility of LBD, given the association between parkinsonism and this disorder [33].

Cognitive testing showed impairments in short-term memory, attention, and executive functions, consistent with neurodegenerative disease profiles [34, 35].

A comprehensive family history revealed no history of dementia or other neurodegenerative disorders. However, the patient's mother reportedly experienced significant anxiety, highlighting the potential relevance of considering psychiatric conditions within the family history [16].

Brain MRI revealed mild to moderate generalized cortical atrophy, with the most pronounced atrophy observed bilaterally in the temporal lobes. Frontal lobe atrophy was also noted, predominantly in the dorsolateral prefrontal cortex. There was no evidence of acute infarction, hemorrhage, or space-occupying lesions.

The MRI findings suggest a neurodegenerative process, particularly a type of dementia. Pronounced atrophy in the temporal lobes, hippocampi, and amygdalae is frequently observed in AD, although it can also manifest in other dementias, like LBD. The negative SPECT DaT scan adds complexity to the diagnostic picture, making PD less likely but not completely ruling out LBD [36, 37]. The patient refused neuropsychological testing, and blood tests did not reveal any specific abnormalities.

Given the patient's symptoms, the clinical presentation, the patient's history and MRI findings, the most likely diagnosis is LBD. This is because, while not exclusive to LBD, the presence of Capgras delusion alongside other symptoms is suggestive of this condition. The patient's history of fragmented sleep, hypersomnolence, and confusion aligns strongly with LBD, where REM sleep behavior disorder and daytime sleepiness are common. Progressive cognitive decline, including memory problems and difficulties with executive function (as suggested by the frontal lobe atrophy), is a core feature of LBD. Furthermore, increased irritability, nervousness, and anxiety, especially in the evening, are consistent with the fluctuating cognitive and behavioral patterns often seen in LBD. The presence of hypnagogic and hypnopompic hallucinations is another strong indicator of LBD. While nonspecific, symptoms, like decreased appetite, nausea, and weight loss can also be associated with LBD. The MRI findings of generalized cortical atrophy with pronounced involvement of the temporal lobes (hippocampi and amygdalae) and frontal lobes are consistent with LBD [38].

While the negative SPECT DaT scan might seem contradictory, it's important to note that this finding doesn't rule out LBD. DaT scans can be negative in a significant proportion of LBD cases, especially in the early stages. Other possible diagnoses to consider, though less likely, include AD and PD dementia. While temporal lobe atrophy is prominent in AD, the presence of Capgras delusion, visual hallucinations, and sleep disturbances points more towards LBD. The negative DaT scan makes PD dementia less likely [39].

The selection of medications and their dosages in this case appear to be thoughtfully tailored to the patient's initial symptoms, their response to treatment, and the potential for side effects.

Lithium sulfate, starting at 83 mg/day, was chosen mainly for mood stabilization, aiming to reduce anxiety, agitation, and sleep issues that likely worsened her delusions and distress [40]. By stabilizing the patient's mood, lithium was expected to create a more favorable psychological environment, potentially reducing the intensity and frequency of her delusions.

Moreover, the choice of lithium was further supported by recent evidence highlighting its neurotrophic and neuroprotective properties. Studies have shown that lithium promotes neurogenesis, enhances synaptic plasticity, and reduces apoptotic signaling in neuronal cells, which could be particularly beneficial in the context of neurodegenerative disorders. Importantly, lithium has demonstrated a relatively low neurotoxic profile compared to other mood stabilizers, making it a safer option for long-term use in patients who may already be vulnerable to neuronal damage.

In summary, the use of lithium in this case was not only justified by its mood-stabilizing effects but also by its potential to offer neuroprotection, addressing both the immediate psychiatric symptoms and the underlying neurodegenerative processes that may be contributing to the patient's condition [19, 20, 41, 42]. Importantly, lithium's minimal impact on executive functions makes it a particularly attractive option for this patient [43–45]. It is important to remember that, in elderly patients, lithium therapy requires caution due to decreased renal clearance, and therefore close monitoring is essential.

*Citalopram*, a selective serotonin reuptake inhibitor, was introduced at a low dose of 10 mg/day, primarily to target the patient's anxiety and panic-agoraphobic symptoms. The choice of citalopram likely stemmed from its extensive use and well-established efficacy in the elderly population, coupled with its favorable cost–benefit ratio within this demographic [46]. Additionally, the potential impact of citalopram on mood and salience was likely considered, particularly given the hypothesis proposed by some researchers that CS may involve alterations in salience processing [47].

*Lurasidone*, an atypical antipsychotic, was incorporated into the treatment plan at an initial dose of 37 mg/day, later titrated to 74 mg/day, with the goal of addressing the patient's delusional thinking. The selection of lurasidone was likely based on its favorable safety profile, excellent blood-brain barrier permeability, and reduced risk of exacerbating extrapyramidal symptoms through its minimal action on the nigrostriatal pathway [48].

The decision to increase Lurasidone to 74 mg/day after the initial visit was likely based on the observed improvements in the patient's overall clinical presentation. The reduction in anxiety, panic-agoraphobic symptoms, and sleep disturbances, along with the decreased emotional distress associated with her delusions, suggested that the medication was having a positive effect.

The continued use of the same medication regimen after 3 months, despite the emergence of mild motor side effects (probably due to both Lithium and Lurasidone), suggests that the overall benefits of the treatment outweighed the risks. The patient exhibited significant improvements in her mood, anxiety, sleep, and ability to engage in daily activities. The slight decline in motor skills, while concerning, was deemed manageable with close monitoring. However, it's important to note that the decision to continue the current treatment regimen should always be made in consultation with the patient and their family, considering their individual needs and preferences. Regular monitoring of both the therapeutic benefits and potential side effects is crucial to ensure the long-term safety and well-being of the patient.

The persistence of Capgras delusion, despite a positive response to other treatments, necessitated a targeted approach. Psychoeducation was provided to the patient and her family, offering clear and accessible information about CS and emphasizing the delusional nature of her beliefs. Supportive therapy, both individual and family-based, was instrumental in helping the patient manage the anxiety and stress associated with the delusion. Additionally, environmental adaptations were implemented to minimize potential triggers for the delusion, such as avoiding direct contradiction of the patient's beliefs or attempts to force recognition of her husband.

## 4. Conclusion

The effective management of CS is a significant clinical challenge that demands a multifaceted, individualized approach. This requires recognizing the intricate interplay of neurological, psychological, and social factors of the disorder. A multidisciplinary team, including neurologists, psychiatrists, psychologists, and other healthcare professionals, is essential to provide comprehensive care.

Early and accurate diagnosis, differentiating CS from conditions, like AD, LBD, and schizophrenia, is crucial for timely intervention and potentially mitigating the progression of underlying neurological issues.

Treatment should be prompt and tailored, integrating pharmacological and psychosocial components, with careful consideration of patient-specific factors and regular monitoring. Beyond addressing delusions, a holistic approach is essential to enhance the patient's well-being and quality of life. Psychosocial interventions, including psychoeducation and supportive therapy, alongside environmental modifications, play a vital role. Ongoing research into the neurobiological mechanisms of CS, including genetic predispositions, refined diagnostic criteria, and targeted therapies, holds promise for improving diagnostics, treatment, and ultimately, outcomes for individuals and families affected by CS.

## Figures and Tables

**Table 1:** Summary table of diagnostic investigations and key findings.

Investigation type	Description	Key findings	Clinical implications
Brain CT scan	Brain CT scan performed to assess brain atrophy	Subtle to mild diffuse cortical atrophy (slightly below normative range for age); possible volume loss in bilateral temporal lobes; minimal prominence of the frontal horns of the lateral ventricles; and no acute findings	Supports the presence of cortical atrophy, suggesting a possible neurodegenerative cause

Brain MRI	Brain MRI performed to assess brain atrophy and rule out other pathologies	Mild-to-moderate generalized cortical atrophy, most pronounced bilaterally in temporal lobes, frontal lobe atrophy, no acute infarction, hemorrhage, or space-occupying lesions	Supports the presence of cortical atrophy, suggesting a possible neurodegenerative cause

SPECT DaT scan	DaT scan to evaluate for possible Lewy body dementia or Parkinson's disease dementia	No lesions indicative of dopaminergic deficits	Rules out dopaminergic deficit, making LBD or PD diagnosis less likely

Neuropsychological testing recommendation	Neuropsychological testing recommended to assess cognitive function	Patient declined testing; bedside cognitive assessments revealed deficits suggestive of impairments in attention, executive function, visuospatial abilities, and memory	Indicates potential cognitive deficits, but not conclusive without formal testing

Blood tests	Blood tests performed to rule out reversible causes of cognitive decline	All within normal limits	Rules out reversible causes of cognitive decline

**Table 2:** Patient timeline: history, diagnosis, and treatment.

Category	Time	Event	Details
Medical history	Prior history	Independent living and career	Held administrative and clerical roles, higher cognitive level previously demonstrated (high school and some college coursework)

Significant event	5 years prior to onset	Husband's death	Patient's husband passed away

Medical history	~1.5 years after husband's death	Moved in with daughter and son-in-law	Required support in daily management and healthcare needs

Symptom onset	~2 years after husband's death, at the age of 70.	Depressive symptoms and panic attacks	Onset of depression and agoraphobia; treated with paroxetine and alprazolam (temporary benefits)

Symptom onset	~1 year prior to onset	Sleep disturbances	Insomnia, night awakenings with confusion, daytime sleepiness, and suspected REM sleep behavior disorder

Symptom onset	6 months before evaluation	Onset of delusion of misidentification	Persistent belief that deceased husband had been replaced by an imposter, accompanied by increased agitation and lack of insight

Diagnostic testing	Initial evaluation	Clinical and cognitive examinations	Resting tremors, slowed movement, postural instability, deficits in attention, short-term memory, and executive functions

Diagnostic testing	Instrumental examinations	Brain imaging	Cortical atrophy (temporal and frontal lobes) and negative SPECT DaT scan (not conclusive for DLB)

Diagnosis	Provisional diagnosis	Lewy body dementia	Key symptoms: Delusion of misidentification, cognitive fluctuations, visual hallucinations, parkinsonism, REM sleep behavior disorder, behavioral, and psychological symptoms

Treatment	Treatment initiation	Pharmacological	Lithium (83 mg/day), Citalopram (10 mg/day), and Lurasidone (37 mg/day and later titrated to 74 mg/day)

Treatment	Environmental adjustments	Support therapies	Psychoeducation for patient and family, and environmental adaptations to reduce delusion triggers, such as simplified home layout, reduced visual clutter, and consistent daily routines

Outcomes	Follow-up	Partial clinical improvement	Improved anxiety and sleep, partial resolution of emotional distress, and resumption of some activities; however, mild decline in motor skills observed

## Data Availability

All data relevant to this case report are included within the manuscript. Additional data are available from the corresponding author upon reasonable request.
